# Phytochemical Diversity and Antimicrobial Potential of Fabaceae Species Occurring in Tamaulipas, Mexico: A Systematic Review

**DOI:** 10.3390/plants15020278

**Published:** 2026-01-16

**Authors:** Paulina Rachel Gutiérrez-Durán, Jorge Víctor Horta-Vega, Fabián Eliseo Olazarán-Santibáñez, Juan Flores-Gracia, Hugo Brígido Barrios-García

**Affiliations:** 1 Instituto Tecnológico de Ciudad Victoria, Tecnológico Nacional de México/Instituto Tecnológico de Ciudad Victoria, Avenida Tecnológico No. 1301, C.P., Ciudad Victoria 87010, Mexico; pgutierrez@uat.edu.mx (P.R.G.-D.); juan.fg@cdvictoria.tecnm.mx (J.F.-G.); 2 Departamento de Bacteriología, Facultad de Medicina Veterinaria y Zootecnia, Universidad Autónoma de Tamaulipas, Carretera Victoria-Mante km. 5 A.P. N° 263, C.P., Ciudad Victoria 87000, Mexico; feolazaran@docentes.uat.edu.mx

**Keywords:** antimicrobial activity, phytochemical compounds, medicinal plants, ethnobotany, extraction techniques

## Abstract

Antimicrobial resistance represents a critical challenge to global public health, driving the search for bioactive compounds in medicinal plants. The Fabaceae family stands out for its chemical richness and pharmacological properties; however, in the state of Tamaulipas, Mexico—an area of high diversity due to its location between the Nearctic and Neotropical regions—this flora remains largely unexplored. The objective of this review was to analyze the global scientific literature on the Fabaceae of Tamaulipas, integrating floristic records, phytochemistry, and antimicrobial activity. Of the 347 species recorded in the state, only 60 have phytochemical studies, and 43 have documented medicinal uses. The results show that extraction methods predominantly use polar solvents to isolate phenolic compounds, flavonoids, and alkaloids, which show efficacy against pathogens such as *Staphylococcus aureus*, *Escherichia coli*, and *Candida albicans*. Despite limited local ethnobotanical documentation, the potential demonstrated by these species in other regions positions Tamaulipas as a strategic reservoir. This review identifies research gaps and emphasizes the need for systematic studies that validate traditional uses and prioritize bioprospecting of the flora of northeastern Mexico for the development of new therapeutic alternatives.

## 1. Introduction

Since ancient times, plants have been used for a wide variety of purposes, including the treatment of diseases due to the presence of bioactive compounds with therapeutic properties, including the control of infectious diseases in animals and humans [1,2]. Both the World Health Organization (WHO) and the World Organization for Animal Health (WOAH) have declared antimicrobial resistance to be one of the greatest threats to public health and animal health worldwide [3,4]. For this reason, there is an urgent need for new alternatives to treat resistant infections, such as natural compounds derived from plants. These organisms produce secondary metabolites, which are potentially promising candidates for drug discovery due to their defense mechanisms against pathogens [5,6,7,8].

The Fabaceae family, comprising 770 genera and approximately 19,500 species worldwide, is recognized for its chemical diversity and medicinal applications. Their high adaptive capacity defines them as a cosmopolitan family. In Mexico, Fabaceae is the second richest family in terms of species, including trees, shrubs, and perennial or annual herbs [9,10,11].

Located in northeastern Mexico, the state of Tamaulipas represents a convergence point between the Nearctic and Neotropical biogeographic regions, giving rise to a complex mosaic of ecosystems. This biodiversity ranges from cloud forests and cold climates to tropical rainforests, arid zones, and scrublands. This ecological transition zone not only fosters a remarkable richness of species and endemism but also harbors unique ecosystems such as coastal dunes and mangroves [9,12].

However, despite this biological value, research efforts in Mexico have historically concentrated in the central and southern parts of the country, resulting in uneven documentation of the flora of the northeast. A critical example is the Altas Cumbres Protected Natural Area in central Tamaulipas, which remains insufficiently studied; in particular, detailed records on the richness and distribution of legumes were lacking, even though they are one of the most representative plant families in the area [9,12,13].

Consequently, the relatively limited documentation of medicinal Fabaceae species in Tamaulipas should not be interpreted as evidence of low biological or pharmacological potential. Rather, it reflects a gap in academic knowledge rather than a lack of biological potential. In contrast, Fabaceae species in states such as Morelos, Hidalgo, and Oaxaca have been studied in relation to their traditional medicinal applications and bioactive compounds [14,15,16,17,18,19]. The medicinal uses of this family traditionally include the treatment of gastrointestinal infections [15,18,20,21,22,23,24,25,26,27,28]; respiratory disorders [24,25,27,29,30,31,32,33]; and skin lesions [14,24,31,32,34,35,36,37,38,39,40]. These therapeutic effects are supported by studies showing significant inhibitory activity against clinically relevant pathogens, including Gram-positive bacteria such as *Staphylococcus aureus* [21,22,24,26,27,32,34,38,41,42,43,44,45]. Gram-negative bacteria such as *Escherichia coli*, *Pseudomonas aeruginosa*, and *Salmonella Typhi* [21,22,32,44,46,47,48], as well as opportunistic fungi such as *Candida albicans* [18,25,27,32,38].

In Tamaulipas, some ethnomedicinal knowledge related to Fabaceae species is limited to local and rural communities, with limited integration regarding their phytochemical and pharmacological composition. Therefore, linking the regional flora with global phytochemical and antimicrobial research is essential to validate traditional knowledge and identify previously untapped therapeutic resources [49].

The objective of this study is to systematically review the Fabaceae species recorded in Tamaulipas, compiling available information on their phytochemical profiles and antimicrobial properties reported both in Mexico and worldwide. This is achieved by integrating local ethnobotanical information with international scientific evidence. This work evaluates the biomedical potential of the Fabaceae species recorded in Tamaulipas, focusing on their antimicrobial activity. By integrating current knowledge about their bioactive compounds, this study serves as a starting point for new research focused on the biotechnological development of the regional flora.

## 2. Materials and Methods

### 2.1. Floristic Dataset and Taxonomic Validation

An assessment of the species richness of the Fabaceae family in the state of Tamaulipas was conducted based on previously documented floristic records. Data on species occurrence were obtained from the article Plantas vasculares de México [13], which reports 347 Fabaceae species in the state. This dataset served as a taxonomic and geographic reference for the review. To ensure the accuracy of the nomenclature, all scientific names were verified using World Flora Online (formerly The Plant List) and compared with the Catalogue of Life [50]. This procedure eliminated synonyms and ensured that all taxa were accepted and recognized in the botanical literature.

### 2.2. Systematic Review Design (PRISMA Declaration)

**Figure 1:**
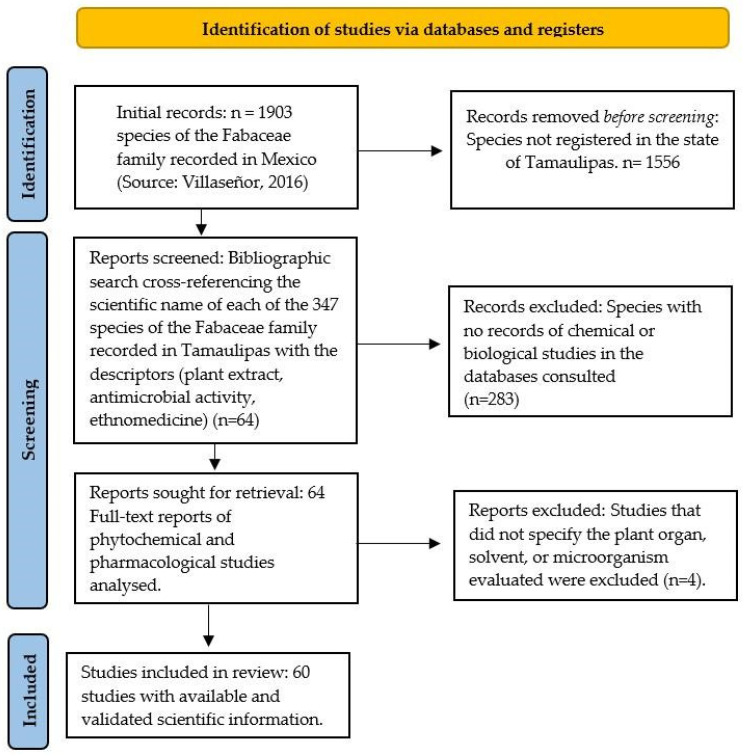
PRISMA diagram of the bibliographic selection process for Fabaceae species in Tamaulipas.

The systematic review was conducted in accordance with the PRISMA 2020 guidelines [51] (Figure 1). The search was performed on platforms such as Google Scholar, Web of Science, PubMed, and SciELO, and the records from 16 countries were included, with no restrictions on language or year of publication. To assess the pharmacological and phytochemical status of the 347 species, each scientific name was used as the main descriptor in combination with the following related keywords: (a) ‘plant extract’, (b) ‘antimicrobial activity’, (c) ‘antibacterial’, (d) ‘bioactive compounds’, and (e) ‘ethnomedicine’ or ‘traditional medicine’. These terms were selected to standardise the search, eliminating non-standardised descriptors such as “phytochemical extracts”. This ensured the use of controlled terminology under international standards, thereby facilitating information retrieval.

### 2.3. Inclusion and Exclusion Criteria

Articles providing specific data on the following were included (1) phytochemical characterization, (2) biological activity against human or animal pathogens, or (3) documented traditional medicinal use. Strict exclusion criteria were applied; articles that did not specify the plant organ used, the solvent used for extraction, or the specific microorganisms analyzed were discarded in order to ensure the integrity of the database. Cases where part of the information was missing were classified as “not reported” (NR) only when the remaining data (e.g., a specific bioactive compound) had high scientific value.

### 2.4. Summary of Data and Regional Context

**Figure 2:**
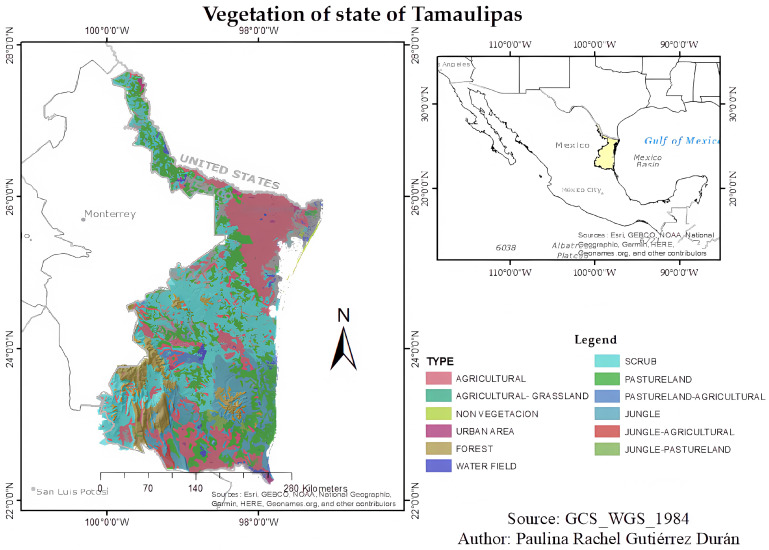
Location of the state of Tamaulipas and types of vegetation.

A total of 107 publications were included, of which 53.27% were published between 2015 and 2025. The data were summarized in tables that included chemical composition, solvents (e.g., methanol, ethanol, water), extraction methods (maceration, Soxhlet), and target microorganisms. The study is focused on species present in Tamaulipas, a strategic state due to its location at the transition between the Nearctic and Neotropical biogeographic regions, covering a topography ranging from sea level to 3100 masl [52] (Figure 2).

## 3. Results and Discussion

### 3.1. Applications in Traditional Medicine

A total of 347 species of the Fabaceae family have been recorded for the state of Tamaulipas, distributed across 81 genera. The family is recognized worldwide for its economic importance, especially in the areas of food production and human and animal health [11,13,53]. This global literature review revealed that only 60 of the 347 species recorded for the state of Tamaulipas have been investigated for phytochemical extraction, and only 43 species have been documented to have traditional medicinal uses worldwide (Table 1). It is important to note that these uses are not necessarily reported for the state of Tamaulipas itself, highlighting the need for research in the region, since Tamaulipas represents a biogeographic transition zone where numerous species are found, where the geographical and climatic conditions of northeastern Mexico allow for the existence of abundant and heterogeneous vegetation, where legumes stand out both for their natural abundance and their socioeconomic relevance, but remain poorly documented ethnobotanically [52,54].

Ethnobotanical studies conducted in local communities have reported on the medicinal use of this family. Hernández-Sandoval and González-Medrano [55] in their work, they reported a list of 58 Fabaceae species, of which 34 had some medicinal use, whether in the bark, root, leaves, seeds, flowers, or exudates. However, they do not emphasize what types of diseases they treat. On the other hand, studies such as those by Jasso-Gandara [56]. also compiled a list of ethnobotanical knowledge in the municipality of Güemez, which includes only one species of Fabaceae. Medellín-Morales and Mora-Ravelo [49] compiled a list of 156 useful plants in the El Cielo Biosphere Reserve, where Fabaceae represent only 4–5% of the usefulness, but they do not mention how many of them have any traditional medicinal use, suggesting that many species remain unexplored locally despite their known pharmacological relevance elsewhere. This discrepancy supports the focus of the present review on Tamaulipas as a region of high biological potential but with limited ethnopharmacological research.

Among the species with medicinal applications listed in Table 1, digestive disorders constitute the most frequent category of use (32%). These species are commonly used to treat symptoms such as diarrhea, vomiting, and stomach pain [53,57]. Diarrhea is the most common ailment. Some of the species studied were *Acaciella angustissima*, *Dalea aurea*, *Gliricidia sepium*, *Grona triflora*, and *Tephrosia cinerea*, from which phenols and flavonoids were extracted, and from *Aeschynomene indica* and *Zornia diphylla*, from which essential oils were extracted [20,43,44,45,46,58,59], followed by general gastrointestinal disorders [42,44].

The second most common category includes dermatological problems (28%), including wound healing, skin infections, and inflammatory disorders. Species of the genus *Neltulma* spp. (formerly *Prosopis* spp.) and species of the genus *Senna* spp. are most commonly used for these conditions. This is attributed to the presence of tannins in the genus *Senna* spp., which have astringent properties, and in the genus *Neltulma* spp. with alkaloids and phenols, which have antimicrobial properties [15,25,26,33,38,40,47,60].

**Table 1:** Species of the Fabaceae family recorded in the state of Tamaulipas, including common names and traditional uses.

Botanical Name	Synonyms	Common Name in México	Traditional Use	References (Study Location)
*Acaciella angustissima* (Mill.) Britton & Rose	*-*	Guajillo	No data recorded	[44] (Queretaro, Mexico)
*Aeschynomene indica* L.	*-*	Not reported	Urticaria, furuncle, nyctalopia, hepatitis, enteritis, and diarrhea.	[20] (Quzhou, China)
*Calliandra tergemina* (L.) Benth.	*-*	Not reported	No data recorded	[61] (Klang, Malaysia)
*Canavalia rosea* (Sw.) DC.	*-*	Frijol de playa	No data recorded	[41] (Crato, Brazil)
*Canavalia villosa* Benth.	*-*	Gallinitas	No data recorded	[62] (Brazil)
*Chamaecrista nictitans* (L.) Moench	*-*	Guajito	Fever and antiviral	[42] (Morelos, Mexico)
*Dalea aurea* Nutt. ex Pursh	*-*	Not reported	Diarrhea, stomach pain, and cramps	[21] (Oklahoma, USA)
*Dalea bicolor* Humb. & Bonpl. ex Willd.	*-*	Escobilla	Gastrointestinal problems, vomiting, and diarrhea	[16] (Hidalgo, Mexico)
*Dalea foliolosa* (Aiton) Barneby	*-*	Almaraduz	Anti-inflammatory and hypoglycemic	[17] (Oaxaca, Mexico)
*Dalea nana* Torr. ex A.Gray	*-*	Trébol enano de pradera	No data recorded	[29] (Arizona, USA)
*Dalea versicolor* Zucc.	*-*	Not reported	No data recorded	[58] (Arizona, USA)
*Desmodium incanum* (Sw.) DC.	*-*	Amor seco	Back pain, colds, and kidney Problems	[30] (Manchester, Jamaica)
*Desmodium scorpiurus* (Sw.) Poir.	*-*	Not reported	Constipation, cough, convulsions, venereal infections, tinea	[63] (Kaduna, Niger)
*Desmodium tortuosum* (Sw.) DC.	*-*	Cadillo	Cardiovascular events	[64] (Ucayali, Peru)
*Ebenopsis ebano* (Berland.) Barneby & J.W.Grimes	*-*	Ébano	No data recorded	[65] (Nuevo Leon, Mexico)
*Enterolobium cyclocarpum* (Jacq.) Griseb.	*-*	Guanacaste	No data recorded	[66] (Oyo, Niger)
*Erythrina herbacea* L.	*-*	Hierba de colorín	No data recorded	[67] (Texas, USA)
*Eysenhardtia platycarpa* Pennell & Saff.	*-*	Not reported	Kidney and gallbladder diseases	[68] (Nuevo Leon, Mexico)
*Gleditsia aquatica* Marshall	*-*	Not reported	No data recorded	[31] (Giza, Egypt)
*Gleditsia triacanthos* L.	*-*	Acacia de tres espinas	Pain, whooping cough, measles, smallpox, skin diseases, asthma	[34] (South Africa)
*Gliricidia sepium* (Jacq.) Kunth	*-*	Cacahuananche	Wounds, diarrhea, repelling mosquitoes, fumigating	[43] (Kerala, India)
*Grona adscendens* (Sw.) H.Ohashi & K.Ohashi	*Desmodium adscendens* (Sw.) DC.	Amor seco	Oral-dental and urogenital problems, and opportunistic infections	[22] (Ibadan, Niger)
*Grona triflora* (L.) H.Ohashi & K.Ohashi	*Desmodium triflorum* (L.) DC.	Hierba cuartillo	Diarrhea, convulsions, tonic, diuretic, and biliary conditions.	[46] (Lucknow, India)
*Haematoxylum brasiletto* H.Karst.	*-*	Madera de Brasil	Oral and kidney infections, hypertension, gastrointestinal disorders, and diabetes.	[69] (Sonora, Mexico)
*Indigofera suffruticosa* Mill.	*-*	Anileira	Healing	[70] (Pernambuco, Brazil)
*Inga vera* Willd.	*-*	Not reported	Treatment of diseases	[23] (Santo Domingo, Dominican Republic)
*Leucaena leucocephala* (Lam.) de Wit	*-*	Not reported	Gastrointestinal	[71] (Ibadan, Niger)
*Lonchocarpus punctatus* Kunth	*-*	Balché	Parasitic	[32] (Yucatan, Mexico)
*Lysiloma acapulcense* (Kunth) Benth.	*-*	Not reported	Respiratory, gastrointestinal, urinary, and skin infections	[72] (Baja California, Mexico)
*Macroptilium lathyroides* (L.) Urb.	*-*	Not reported	No data recorded	[73] (Chennai, India)
*Mimosa malacophylla* A.Gray	*-*	Not reported	Diuretic and kidney stones	[74] (Nuevo Leon, Mexico)
*Mucuna pruriens* (L.) DC.	*-*	Mucuna	Purgative and diuretic	[24] (Osun, Niger)
*Neltuma glandulosa* (Torr.) Britton & Rose	*Prosopis glandulosa* Torr.	Mesquite dulce	Gastrointestinal, rashes, eye infections, hernias, skin conditions, sore throat	[33] (Nevada, USA)
*Neltuma juliflora* (Sw.) Raf.	*Prosopis juliflora* (Sw.) DC.	Mesquite	Colds, diarrhea, flu, hoarseness, inflammation, measles, sore throat, liver and eye problems	[25] (Bushehr, Iran)
*Neltuma laevigata* (Humb. & Bonpl. ex Willd.) Britton & Rose	*Prosopis laevigata* (Humb. & Bonpl. ex Willd.) M.C.Johnst.	Mesquite	Skin, gastrointestinal, and respiratory diseases	[60] (Zapotitlan Salinas, Mexico)
*Neptunia oleracea* Lour.	*-*	Mimosa de agua	Diabetes mellitus, inflammation, and fever	[35] (Selangor, Malaysia)
*Pachyrhizus erosus* (L.) Urb.	*-*	Jícama	Skin rashes	[14] (Morelos, Mexico)
*Parkinsonia aculeata* L.	*-*	Escoba	Skin and urinary tract infections	[75] (Maharashtra, India)
*Parkinsonia florida* (Benth. ex A.Gray) S.Watson	*-*	Palito azul verdoso	No data recorded	[18] (Sonora, Mexico)
*Parkinsonia praecox* (Ruiz & Pav.) Hawkins	*-*	Palo brea	Gastrointestinal, antitussive, wound healing, headaches, earaches, and scorpion stings	[19] (Oaxaca, Mexico)
*Phaseolus coccineus* L.	*-*	Ayocote	No data recorded	[76] (Dali, China)
*Phaseolus lunatus* L.	*-*	Habas	Food	[77] (Machala, Ecuador)
*Phaseolus vulgaris* L.	*-*	Frijoles	Food	[36] (Giza, Egypt)
*Pithecellobium dulce* (Roxb.) Benth.	*-*	Jungli Jalebi	Earache, leprosy, peptic ulcer, and toothache	[37] (Haryana, India)
*Rhynchosia minima* (L.) DC.	*-*	Frijolillo	Skin conditions and to relieve boils.	[78] (Harare, Zimbabwe)
*Senegalia berlandieri* (Benth.) Britton & Rose	*-*	Espino	No data recorded	[79] (Texas, USA)
*Senegalia greggii* (A.Gray) Britton & Rose	*-*	Tesota	No data recorded	[79] (Texas, USA)
*Senna crotalarioides* (Kunth) H.S.Irwin & Barneby	*-*	Not reported	Inflammation	[80] (San Luis Potosi, Mexico)
*Senna hirsuta* (L.) H.S.Irwin & Barneby	*-*	Cuajillo	Hypertension, dropsy, diabetes, fevers, bile, rheumatism, tinea, and eczema	[48] (Uyo, Niger)
*Senna obtusifolia* (L.) H.S.Irwin & Barneby	*-*	Tasba	Eye infection and laxative	[47] (Yola, Niger)
*Senna occidentalis* (L.) Link	*-*	Candelilla pequeña	Malaria and trypanosomiasis	[40] (Minna, Niger)
*Senna septemtrionalis* (Viv.) H.S.Irwin & Barneby	*-*	Cafecillo	Diuretic, anti-inflammatory, laxative, expectorant, and fungicide, fever, burns, cholera, hemorrhoids, pain, gastroenteritis.	[38] (Guanajuato, Mexico)
*Senna wislizeni* (A.Gray) H.S.Irwin & Barneby	*-*	Carrozo	Laxative properties, skin and parasitic diseases	[15] (Morelos, Mexico)
*Sophora tomentosa* L.	*-*	Not reported	Cholera, diarrhea, gastrointestinal antidote	[27] (Giza, Egypt)
*Tephrosia cinerea* (L.) Pers.	*-*	Bardana medicinal	Diarrhea, diuretic, bronchitis, asthma, inflammation	[59] (Chamrajanagar, India)
*Vachellia farnesiana* (L.) Wight & Arn.	*-*	Huizache	No data recorded	[79] (Texas, USA)
*Vachellia rigidula* (Benth.) Seigler & Ebinger	*-*	Chaparro prieto	No data recorded	[79] (Texas, USA)
*Vigna luteola* (Jacq.) Benth.	*-*	Porotillo	No data recorded	[81] (Nantou, Taiwan)
*Vigna vexillata* (L.) A.Rich.	*-*	Bejuco pato	No data recorded	[39] (Nantou, Taiwan)
*Zapoteca portoricensis* (Jacq.) H.M.Hern.	*-*	Palo blanco	Convulsions, constipation, skin infections	[28] (Abakaliki, Niger)
*Zornia diphylla* (L.) Pers.	*-*	Raíz de víbora	Diarrhea and venereal diseases	[45] (Kerala, India)

In relation to Table 1, it is important to clarify that species lacking common names or documented traditional uses do not necessarily lack medicinal value on a global scale. Rather, these absences reflect a lack of specific ethnobotanical records, highlighting gaps in regional scientific documentation.

Overall, these findings demonstrate that Fabaceae species found in Tamaulipas have documented medicinal relevance in other regions, reinforcing the importance of this state as a priority area for future ethnobotanical, phytochemical, and pharmacological studies.

### 3.2. Phytochemical Extraction Methods and Target Compounds

This section analyzes extraction strategies in relation to the physicochemical properties of secondary metabolites. In the studies reviewed, extraction methods were selected based on the chemical nature and stability of the metabolites to be extracted [82], as each extraction method has different principles, strengths, and specific limitations that affect the yield and composition of the extract [83].

Maceration was the most widely used technique (62%) due to its simple methodological flexibility and, above all, its ability to protect heat-sensitive compounds such as flavonoids [83]. It was associated with the recovery of flavonoids, tannins, and other polyphenols mainly from leaves, which accumulate these metabolites as a method of defense and protection, as they are exposed to UV radiation, herbivores, pathogens, and environmental stress [84]. On the other hand, Soxhlet extraction (10.8%) and reflux extraction (7.7%) were preferred in studies focusing on thermostable or less polar compounds, including certain alkaloids and terpenoids. Although these methods enable higher extraction yields under controlled conditions, their use is limited by the thermal sensitivity of many metabolites [83,85,86]. Studies focusing on volatile components, particularly essential oils, employed hydrodistillation (4.6%), which remains the standard method for terpene-rich fractions. The genera *Senna*, *Dalea*, *Rhynchosia*, and *Zornia* are reliable sources of essential oils when appropriate methods are used.

The extraction methods summarized in Table 2 are reported as methodological tools selected to achieve specific phytochemical objectives. The reported variability highlights the adaptability of extraction protocols to the specific objectives of each compound.

#### 3.2.1. Solvents

The choice of solvent for phytochemical extraction is essential and depends both on the chemical characteristics of the target compounds and on the specific plant material used. The type of metabolite being sought helps determine the most suitable solvent, as each compound dissolves more effectively in solvents of different polarity [85]. Polar compounds are best extracted with solvents such as methanol or ethanol, which were mainly used for the extraction of phenols and flavonoids (28%). Methanol was the most commonly used solvent due to its intermediate polarity and broad extraction capacity [87]. However, several studies increasingly prioritized ethanol and aqueous extracts, especially in bioactivity assays, due to their lower toxicity and greater relevance for pharmacological and ethnomedical applications [86,87,88]. Nonpolar metabolites are commonly extracted with solvents such as hexane and ethyl acetate to isolate essential oils, flavonols, and isoflavones (4.5%). The use of ethyl acetate reflects a methodological shift toward solvents with lower environmental and health risks compared to petroleum-based alternatives [89].

#### 3.2.2. Plant Organs and Biological Forms

The predominance of leaves as the extracted organ (53%) is consistent with their high biosynthetic activity; leaves accumulate the highest concentration of alkaloids and polyphenols, which act as a defense mechanism against local herbivores, a characteristic that humans exploit for therapeutic antimicrobial purposes [84]. Multiple studies included in Table 2 report higher antioxidant and antimicrobial activity in leaf extracts compared to other organs, supporting their frequent selection [90,91,92]. The seeds, although less studied (13.8%), are mainly studied for their oil content and antioxidant properties [93].

The biological form categories of the plant species included shrubs, trees, herbs, and climbers. Herbaceous and tree species were the most frequently studied, accounting for 36% and 31% of the records, respectively. These categories are most commonly collected and analyzed in ethnobotanical studies due to their abundance, availability, and ease of collection in the field, making them more accessible for research. Shrub species followed in lower proportion, and climbers were the least represented [90,94].

### 3.3. Isolated Compounds

Table 3 summarises the bioactive compounds isolated from the Fabaceae species included in this review, highlighting both their chemical diversity and the biological activities described. The most frequently identified metabolites are phenolic compounds and flavonoids (56%). These compounds were recovered from 36 Fabaceae species and have been associated with antioxidant, antibacterial, and antifungal activities, particularly against *Staphylococcus aureus*, *Escherichia coli*, and *Candida albicans* [95,96]. The most representative species in terms of phenol/flavonoid isolation and antioxidant activity were: *Acacia angustissima*, *Desmodium tortuosum*, *Vigna luteola*, and *Senna pendula*.

Several studies also reported the isolation of alkaloids and showed a strong association with antimicrobial (65.4%) and cytotoxic (30.8%) activity. The species related to these activities were: *Gleditsia triacanthos*, *Inga vera*, *Neltuma glandulosa*, and *Senna occidentalis* in terms of alkaloid and tannin isolation; while the species *Lonchocarpus punctatus*, *Macroptilium lathyroides*, *Parkinsonia praecox*, and *Sophora tomentosa* showed anticancer activities. Essential oils, although less frequent (10%), also showed antimicrobial activity.

Tannins were identified in 34.4% of the species analyzed, mainly associated with antimicrobial activity, followed by anti-inflammatory and antiparasitic effects. These results reinforce the role of tannins as key metabolites in the chemical defense of plants and their pharmacological relevance [84,97]. In addition, 26.6% of the species analyzed contained saponins and 32.8% contained terpenoids. Of these, approximately 30% were associated with anti-inflammatory activity and 14–24% with antiparasitic effects, supporting the role of these metabolites in modulating inflammatory processes and defending against parasites. Isoflavones and lectins, especially those from seed extracts, were associated with antioxidant and immunomodulatory activities.

Overall, the compounds summarized in Table 3 demonstrate that Fabaceae species harbor a broad spectrum of structurally diverse metabolites whose biological activities support both their traditional uses and their potential for drug development.

It is important to note that not all studies included in this section evaluated antimicrobial activity. Several investigations focused on the anticancer and cytotoxic potential of the isolated compounds, given their clinical relevance. In this context, alkaloids, isoflavones, and triterpenoids have been reported to exhibit cytotoxic effects against various cancer cell lines [5,32,39,80,81].

#### Biological Properties and Mechanisms of Action of the Main Isolated Compounds

The medicinal potential of Fabaceae species depends on their chemical diversity. This review demonstrates that antimicrobial action is determined by specific groups of metabolites that act precisely on different microorganisms (Table 3).

Phenols and flavonoids were the most common compounds isolated from Fabaceae species and showed a wide range of biological activities on different microorganisms. The antibacterial mechanisms of flavonoids include inhibition of nucleic acid synthesis, disruption and damage of the bacterial cytoplasmic membrane, and inhibition of biofilm formation [98]. For example, phenols and flavonoids such as protocatechuic acid and catechin isolated from *Acaciella angustissima* at a dose of 200 mg/mL showed inhibitory effects against *Rhizoctonia solani*, *Fusarium oxysporum*, and *Phytophthora capsici* [44]. Similarly, flavonols such as kaempferol and quercetin isolated from *Calliandra tergemina* showed antibacterial effects against *Staphylococcus aureus* at concentrations of 1 mg/100 µL [63]. Flavonoids from *Dalea nana* and *Dalea versicolor* showed antimicrobial activity against *Staphylococcus aureus*, *Bacillus cereus*, *Candida albicans*, and *Cryptococcus neoformans* [29].

Alkaloids act mainly on the bacterial cell membrane, causing structural leaks and death. They are particularly effective against Gram-positive strains, but have a broad spectrum of activity [99]. They have been isolated in several genera, including *Desmodium*, *Erythrina*, *Neltuma*, and *Lonchocarpus*. Alkaloids from *Desmodium scorpiurus* showed antibacterial activity against *Escherichia coli*, *Pseudomonas aeruginosa*, and *Streptococcus pyogenes* [63], while alkaloids isolated from *Neltuma glandulosa* demonstrated antiparasitic and antifungal activity against *Leishmania donovani*, *Plasmodium falciparum*, and *Cryptococcus neoformans* [33]. On the other hand, alkaloids and related compounds isolated from *Lonchocarpus punctatus* were evaluated mainly for their anticancer activity, without microbial assays, highlighting their clinical relevance despite the absence of antimicrobial data [32].

Tannins and saponins showed remarkable antimicrobial and cytotoxic effects. Tannins exert antimicrobial effects by forming complexes with bacterial proteins and enzymes, while saponins act as detergents that alter the membrane or induce cytotoxicity [46,100]. Tannins isolated from *Lysiloma acapulcense* inhibited the growth of bacteria such as *E. coli*, *P. aeruginosa*, *S. aureus*, and the fungus *Candida albicans* at concentrations of 2.5 µg/mL to 5.0 µg/mL [72]. Saponins from *Gleditsia aquatica* showed cytotoxic activity, supporting their potential anticancer relevance, although no microbial assays were performed [31]. In *Senna obtusifolia*, the presence of tannins and saponins contributes to a broad spectrum against the bacteria *Neisseria gonorrhoeae*, *S. aureus*, and *S. aerugenosa* [47].

Lectins inhibit growth by recognizing specific carbohydrates on microbial surfaces, often agglutinating cells or triggering immune responses [101]. The *Canavalia* and *Phaseolus* species showed antifungal and antibacterial activity. Lectins from *Canavalia rosea* inhibited the growth of *Candida albicans* [41], while those isolated from *Phaseolus vulgaris* and *Phaseolus coccineus* demonstrated antibacterial and antifungal activity against various pathogenic bacteria and fungi [36,76].

Terpenoids and essential oils, due to their lipophilic nature, easily penetrate the lipid bilayer of membranes and exhibit strong antimicrobial activity [102]. They were mentioned less frequently but showed strong antimicrobial potential. The essential oils of *Aeschynomene indica* and *Leucaena leucocephala* showed antibacterial and antifungal activities against *Staphylococcus aureus*, *Escherichia coli*, and *Candida albicans* [20,71]. Terpenoids isolated from *Parkinsonia praecox* showed both antibacterial activity against *Listeria monocytogenes* and anticancer potential [19].

### 3.4. Microorganisms Evaluated and Antimicrobial Assessment

The studies reviewed evaluated a broad range of microorganisms, including Gram-positive and Gram-negative bacteria, yeasts, filamentous and phytopathogenic fungi, as well as clinically relevant parasites (Table 3). This range indicates that the biological activity of Fabaceae secondary metabolites extends beyond antibacterial effects. Among Gram-positive bacteria, *Staphylococcus aureus* was the most frequently tested species.

Several studies reported inhibitory activity associated with phenols, flavonoids, alkaloids, lectins, and essential oils [103,104]. Essential oils from *Aeschynomene indica* inhibited *S. aureus* at concentrations of 0.312–0.625 mg/mL [20] while metabolites from *Parkinsonia florida* and *Dalea* spp. showed activity between 10 and 2000 µg/mL [18,61]. Alkaloids isolated from *Erythrina herbacea* exhibited dosages of 6.25–50 µg/mL [67].

Gram-negative bacteria, particularly *Escherichia coli* and *Pseudomonas aeruginosa*, were also commonly evaluated but generally required higher inhibitory concentrations. Extracts containing phenols and alkaloids from *Desmodium scorpiurus* inhibited *E. coli* at concentrations up to 200 mg/mL [63]. In contrast, tannin-rich extracts from *Lysiloma acapulcense* showed activity against *E. coli*, *P. aeruginosa*, and *S. aureus* at doses of 2.5–5.0 µg/mL [72]. The lower susceptibility of Gram-negative bacteria is consistent with the presence of an outer membrane that restricts the penetration of polar and high-molecular-weight compounds [105,106]. Terpenoids such as carvacrol, limonene, and linalool were active against both bacterial groups, although *Pseudomonas* spp. and *Streptococcus* spp. showed greater resistance [104].

Fungal microorganisms accounted for a considerable proportion of the studies evaluated. Yeasts such as *Candida albicans* and *Cryptococcus neoformans* were inhibited by lectins, flavonoids, and essential oils. Lectins from *Canavalia rosea* inhibited the growth of *C. albicans* [41], while flavonoids isolated from *Dalea nana* showed activity against *C. albicans* and *C. neoformans* [29]. Filamentous and phytopathogenic fungi, including *Fusarium oxysporum*, *Rhizoctonia solani*, *Colletotrichum gloeosporioides*, and *Phytophthora capsici*, were sensitive to extracts rich in phenols and flavonoids. Extracts from *Acaciella angustissima* inhibited *R. solani* and *F. oxysporum* [44], while isoflavones from *Pachyrhizus erosus* showed antifungal activity against *F. oxysporum* and *R. stolonifer* [14].

In addition to bacteria and fungi, some studies have examined antiparasitic activity, highlighting the clinical value of the isolated compounds. Isoflavones from *Dalea aurea* exhibited antiamebic effects against *Naegleria fowleri* [21] and alkaloids from *Neltuma glandulosa* showed efficacy against *Leishmania donovani* and *Plasmodium falciparum* [33]. These results emphasize antiparasitic action beyond antimicrobial screening.

The techniques used to evaluate plant extracts are fundamental, as they not only demonstrate the antimicrobial activity of species in the Fabaceae family. Ten different antimicrobial evaluation techniques were identified in the literature reviewed, and approximately 70% of the studies focused on bacterial assays. Disc diffusion and well diffusion methods were the most widely used, accounting for 47.17% of the studies, followed by broth microdilution and its variants (19.81%).

This methodological trend highlights the exploratory nature of various phytochemical studies, where diffusion techniques are used as an initial strategic screening method to detect antimicrobial activity in crude extracts or complex fractions. However, diffusion methods have limitations that affect the interpretation of antimicrobial activity. These assays only provide qualitative or semi-quantitative data, without accurately determining minimum inhibitory concentrations [107].

The clinical potential of Fabaceae lies in their multifunctional action against bacterial, fungal, and parasitic pathogens. To rigorously assess this bioactivity, it is essential to integrate the analysis of microbial models with the testing techniques used; only through this joint approach is it possible to contextualize the efficacy of their metabolites and guarantee the scientific validity of the findings. Thus, considering the diversity of pathogens evaluated allows us to gauge the true therapeutic scope of their compounds, avoiding underestimating the biological richness documented in the literature.

## 4. Conclusions

This global bibliographic review integrates floristic records, phytochemical evidence, ethnobotanical information, and antimicrobial data for Fabaceae species occurring in the state of Tamaulipas, Mexico, to identify knowledge gaps and research opportunities relevant to the discovery of plant-derived therapeutic agents. The analysis reveals a marked imbalance between phytochemical evaluation and documented traditional use: although approximately 19% of Fabaceae species in the region have been investigated phytochemically, only 13.3% have recorded ethnomedical applications. This disparity indicates that several native species containing confirmed bioactive metabolites remain largely unexplored from an ethnopharmacological perspective.

The main contribution of this review is the systematic integration of floristic records and phytochemical evidence, aiming for a more comprehensive assessment of the pharmacological potential of Fabaceae species present in Tamaulipas. While the state has a total of 347 species in this family, only 60 species have available information on their chemical extracts, highlighting a significant knowledge gap. The inclusion of species without traditional records or prior studies of antimicrobial activity is relevant, as the presence of secondary metabolites extracted from these species suggests an as-yet-unexplored bioactive potential.

Furthermore, the more than 280 unstudied species represent considerable value with potential therapeutic applications, and their lack of study should not be interpreted as an absence of pharmacological value. This review, therefore, not only synthesizes the available information but also underscores the need to expand phytochemical and pharmacological studies within this family, providing a starting point for prioritizing future research and strengthening knowledge about the medicinal potential of Fabaceae in the region.

## Figures and Tables

**Table 2:** Register of solvents and extraction methods applied to species of the Fabaceae family in Tamaulipas.

Botanical Name	Biological Form	Organ Used	Extraction Technique	Solvent	References
*Acaciella angustissima* (Mill.) Britton & Rose	Shrubby	Seeds	Maceration and Soxhlet method	Methanol	[44]
*Aeschynomene indica* L.	Shrubby	Leaves and stems	Hydro-distillation method	Distilled water	[20]
*Calliandra tergemina* (L.) Benth.	Shrubby	Leaves	Maceration method	Hexane, dichloromethane, ethyl acetate, methanol, and distilled water	[61]
*Canavalia rosea* (Sw.) DC.	Herbaceous	Seeds	Purification	Distilled water	[41]
*Chamaecrista nictitans* (L.) Moench	Herbaceous	Aerial parts	Maceration method	Ethyl acetate	[42]
*Dalea aurea* Nutt. ex Pursh	Herbaceous	Whole plant	Maceration method	Methanol	[21]
*Dalea bicolor* Humb. & Bonpl. ex Willd.	Shrubby	Whole plant	Maceration method	Distilled water and methanol	[16]
*Dalea foliolosa* (Aiton) Barneby	Herbaceous	Leaves	Hydro-distillation method	Distilled water	[17]
*Dalea nana* Torr. ex A.Gray	Herbaceous	Roots and aerial parts	Maceration method	Methanol	[29]
*Dalea versicolor* Zucc.	Herbaceous	Whole plant	Maceration method	Ethanol and methanol	[29]
*Desmodium incanum* (Sw.) DC.	Herbaceous	Leaves and flowers	Maceration method	Methane and distilled water	[30]
*Desmodium scorpiurus* (Sw.) Poir.	Herbaceous	Aerial parts	Soxhlet method	Petroleum alcohol (60–80 °C), chloroform, and methanol.	[63]
*Desmodium tortuosum* (Sw.) DC.	Shrubby	Stems and leaves	Reflux method	Distilled water	[64]
*Ebenopsis ebano* (Berland.) Barneby & J.W.Grimes	Arboreal	Seeds	Maceration method	Distilled water and methanol	[65]
*Enterolobium cyclocarpum* (Jacq.) Griseb.	Arboreal	Leaves	Reflux method	Ethanol	[66]
*Erythrina herbacea* L.	Shrubby	Roots	Maceration method	Ethyl acetate, n-hexane, acetone	[67]
*Eysenhardtia platycarpa* Pennell & Saff.	Arboreal	Branches and leaves	Maceration method	Distilled water, ethanol, and methanol	[68]
*Gleditsia aquatica* Marshall	Arboreal	Fruit	Maceration method	Ethanol	[31]
*Gleditsia triacanthos* L.	Arboreal	Leaf, seeds, and stems	Maceration method	Methanol	[34]
*Gliricidia sepium* (Jacq.) Kunth	Arboreal	Leaf	Maceration method	Ethanol	[43]
*Grona adscendens* (Sw.) H.Ohashi & K.Ohashi	Herbaceous	Root	Maceration method	Methanol	[22]
*Grona triflora* (L.) H.Ohashi & K.Ohashi	Herbaceous	Whole plant	Maceration method	Distilled water and methanol	[46]
*Haematoxylum brasiletto* H.Karst.	Arboreal	Stems	Maceration method	Methanol	[69]
*Indigofera suffruticosa* Mill.	Arboreal	Leaf	Maceration method	Acetone, ether, and chloroform	[70]
*Inga vera* Willd.	Arboreal	Bark	Maceration method	Ethanol	[23]
*Leucaena leucocephala* (Lam.) de Wit	Arboreal	Seeds	Maceration method	Hexane	[71]
*Lonchocarpus punctatus* Kunth	Arboreal	Inflorescence	Maceration method	Ethanol	[32]
*Lysiloma acapulcense* (Kunth) Benth.	Arboreal	Stems and root	Maceration method	Distilled water	[72]
*Macroptilium lathyroides* (L.) Urb.	Herbaceous	Leaf	Maceration method	Distilled water	[73]
*Mimosa malacophylla* A.Gray	Shrubby	Leaf	Maceration method	Ethanol	[74]
*Mucuna pruriens* (L.) DC.	Climbing	Leaf	Maceration method	Methanol	[24]
*Neltuma glandulosa* (Torr.) Britton & Rose	Arboreal	Leaf	Percolation method	Ethanol	[33]
*Neltuma juliflora* (Sw.) Raf.	Arboreal	Seeds	Maceration method	Distilled water, methanol, and ethyl acetate	[25]
*Neltuma laevigata* (Humb. & Bonpl. ex Willd.) Britton & Rose	Arboreal	Leaf	Maceration method	Methanol	[60]
*Neptunia oleracea* Lour.	Herbaceous	Leaf and stem	Soxhlet method	Methanol	[35]
*Pachyrhizus erosus* (L.) Urb.	Herbaceous	Seeds	Maceration method	Hexane, dichloromethane, and acetone	[14]
*Parkinsonia aculeata* L.	Shrubby	Leaf	Soxhlet method	Ethanol, methanol	[75]
*Parkinsonia florida* (Benth. ex A.Gray) S.Watson	Arboreal	Leaf	Reflux method	Distilled water	[18]
*Parkinsonia praecox* (Ruiz & Pav.) Hawkins	Arboreal	Bark	Maceration method	Methanol	[19]
*Phaseolus coccineus* L.	Herbaceous	Seeds	Purification	Distilled water	[76]
*Phaseolus lunatus* L.	Herbaceous	Seeds	Purification	Distilled water	[77]
*Phaseolus vulgaris* L.	Herbaceous	Seeds	Purification	Ammonium sulfate	[36]
*Pithecellobium dulce* (Roxb.) Benth.	Arboreal	Leaf	Maceration method	Benzene, chloroform, acetone, methanol, and distilled water	[37]
*Rhynchosia minima* (L.) DC.	Climbing	Leaf	Hydro-distillation method	Distilled water	[78]
*Senegalia berlandieri* (Benth.) Britton & Rose	Shrubby	Leaf	Soxhlet method	Ethanol, chloroform, ethyl acetate	[79]
*Senegalia greggii* (A.Gray) Britton & Rose	Shrubby	Leaf	Soxhlet method	Ethanol, chloroform, ethyl acetate	[79]
*Senna crotalarioides* (Kunth) H.S.Irwin & Barneby	Shrubby	No data recorded	Reflux method	Chloroform	[80]
*Senna hirsuta* (L.) H.S.Irwin & Barneby	Shrubby	Fruit	Hydro-distillation method	Distilled water	[48]
*Senna obtusifolia* (L.) H.S.Irwin & Barneby	Herbaceous	Leaf	Reflux method	Acetone, hexane, dichloromethane, methanol	[47]
*Senna occidentalis* (L.) Link	Herbaceous	Leaf	Maceration method	Methanol	[40]
*Senna septemtrionalis* (Viv.) H.S.Irwin & Barneby	Shrubby	Aerial parts	Maceration method	Ethanol	[38]
*Senna wislizeni* (A.Gray) H.S.Irwin & Barneby	Shrubby	Whole plant	Maceration method	Methanol and hexane	[15]
*Sophora tomentosa* L.	Shrubby	Leaf	Maceration method	Petroleum ether	[27]
*Tephrosia cinerea* (L.) Pers.	Herbaceous	Leaf	Maceration method	Ethyl acetate, acetone, petroleum ether	[59]
*Vachellia farnesiana* (L.) Wight & Arn.	Arboreal	Leaf	Soxhlet method	Ethanol, chloroform, ethyl acetate	[79]
*Vachellia rigidula* (Benth.) Seigler & Ebinger	Shrubby	Leaf	Soxhlet method	Ethanol, chloroform, ethyl acetate	[79]
*Vigna luteola* (Jacq.) Benth.	Herbaceous	Whole plant	Maceration method	Methanol	[81]
*Vigna vexillata* (L.) A.Rich.	Herbaceous	Whole plant	Maceration method	Methanol, chloroform, and distilled water	[39]
*Zapoteca portoricensis* (Jacq.) H.M.Hern.	Shrubby	Leaf	Maceration method	Water, methanol, ethyl acetate, diethyl ether	[28]
*Zornia diphylla* (L.) Pers.	Herbaceous	Whole plant	Hydro-distillation method	Distilled water	[45]

**Table 3:** Isolated compounds, bioactive properties, and effects on microorganisms of species of the Fabaceae family.

Botanical Name	Isolated Compounds	Bioactive Properties	Effect on Microorganisms	Study/Dose Used	References
*Acaciella angustissima* (Mill.) Britton & Rose	Phenols and flavonoids	Antioxidants, antimutagenic, antidiabetic, anticancer, and anti-inflammatory.	*Rhizoctonia solani*, *Fusarium oxysporum* and *Phytophtora capsici*	Dextrose potato agar culture (200 mg/mL)	[44]
*Aeschynomene indica* L.	Essential oils	Antibacterial, antioxidant, and cytotoxic	*Staphylococcus aureus* and *Bacillus subtilis*	Broth dilution (0.312–0.625 mg/mL)	[20]
*Calliandra tergemina* (L.) Benth.	Flavonol	Antioxidant	*Staphylococcus aureus*	Disc diffusion (1.00 mg/100 µL)	[61]
*Canavalia rosea* (Sw.) DC.	Lectins	Not reported	*Candida albicans*	Microdilution (512 to 0.5 µg/mL)	[41]
*Chamaecrista nictitans* (L.) Moench	Flavonoids, ellagic acid, and proanthocyanidin oligomers	Anthelmintic, antioxidant, and prebiotic	*Haemonchus contortus*	Ovicidal activity (2134 and 601 µg/mL)	[42]
*Dalea aurea* Nutt. ex Pursh	Isoflavones	Anti-amebic	*Naegleria fowleri*	In vitro assay (10 µg/mL)	[21]
*Dalea bicolor* Humb. & Bonpl. ex Willd.	Crude extracts	Not reported	*Salmonella choleraesuis*, *Escherichia coli*, *Staphylococcus aureus* *Bacillus subtilis* *Pseudomonas aeruginosa Salmonella typhi*	Broth dilution (50 and 100 mg/mL)	[16]
*Dalea foliolosa* (Aiton) Barneby	Monoterpenes, sesquiterpenes, and aliphatic hydrocarbons	Antioxidant, anti-a-glucosidase	*Pseudomonas syringae*	Microdilution (35–155 μg mL^−1^)	[17]
*Dalea nana* Torr. ex A.Gray	Flavonoids	Antimicrobial	*Cryptococcus neoformans*, *Staphylococcus aureus*, *Candida albicans.*	Microdilution (6.7–37.0 μM)	[29]
*Dalea versicolor* Zucc.	Flavonoids	Antimicrobial	*Staphylococcus aureus* and *Bacillus cereus*	Microdilution (10–30 µg/mL)	[29]
*Desmodium incanum* (Sw.) DC.	Flavonoids, alkaloids, and tannins	Antimicrobial	*Staphylococcus aureus*, *Streptococcus* and *Klebsiella Pneumoniae*	Well diffusion (5–100 mg/dL)	[30]
*Desmodium scorpiurus* (Sw.) Poir.	Alkaloids, saponins, glycosides, steroids, and flavonoids	Antibacterial	*Pseudomonas aeruginosa*, *Escherichia coli* and *Streptococcus pyrogenes*	Broth dilution (200 mg/mL)	[63]
*Desmodium tortuosum* (Sw.) DC.	Phenols, flavonoids, carotenoids	Antioxidant	Cell model	Microdilution (200 µg/mL)	[64]
*Ebenopsis ebano* (Berland.) Barneby & J.W.Grimes	Phenols	Antimicrobial	*Escherichia coli*, *Salmonella enterica* and *Candida albicans*	Colorimetric assay (125–500 mg/mL)	[68]
*Enterolobium cyclocarpum* (Jacq.) Griseb.	Phenols	Antimicrobial	*Serratia liquefaciens* and *Staphylococcus warneri*	Disc diffusion (10 μL)	[66]
*Erythrina herbacea* L.	Alkaloids	Not reported	*Staphylococcus aureus*	Microdilution (6.25–50 μg/mL)	[67]
*Eysenhardtia platycarpa* Pennell & Saff.	Flavonoids, phenols, and coumarins	Anti-inflammatory, antifungal	Cell model	No data recorded	[68]
*Gleditsia aquatica* Marshall	Saponins	Cytotoxic	Cell model	No data recorded	[31]
*Gleditsia triacanthos* L.	Phenols, flavonoids, tannins, saponins, alkaloids, terpenoids, steroids, cardiac glycosides	Analgesic, anti-inflammatory, hepatoprotective, and antimicrobial activity	*Proteus* spp., *Streptococcus* spp., *Escherichia coli* and *Enterobacter* spp. *C. albicans.*	Well diffusion (1000, 500, 250, 125, 62.5 and 31. 25 μg/mL)	[34]
*Gliricidia sepium* (Jacq.) Kunth	Glycosides, phytosterols, alkaloids, oils, saponins, phenols, and flavonoids	Antibacterial, antifungal, antiviral, and antioxidant	*Escherichia coli* and *Pseudomonas aeroginosa*	Disc diffusion (0.1 g/1 mL)	[43]
*Grona adscendens* (Sw.) H.Ohashi & K.Ohashi	Tannins, saponins, alkaloids, and flavonoids	Antimicrobial	*Staphylococcus aureus*, *Candida albicans*	No data recorded (0.25–0.50 mg/mL)	[22]
*Grona triflora* (L.) H.Ohashi & K.Ohashi	Alkaloids, steroids, tannins, saponins, and flavonoids	Antispasmodic, sympathomimetic, central nervous system stimulant, and diuretic	*Staphylococcus aureus*, *Micrococcus luteus*, *Bacillus pumilus*, *Pseudomonas aeruginosa*, *Pseudomonas fluorescens*, *Escherichia coli*	Disc diffusion (50 and 100 μg/mL)	[46]
*Haematoxylum brasiletto* H.Karst.	Flavonoids	Antimicrobial	*Candida albicans*	Disc diffusion (8.7 to 128 μg/mL)	[69]
*Indigofera suffruticosa* Mill.	Alkaloids, flavonoids, phenylpropanoids, triterpenoids, volatile oils	Anti-inflammatory and anticonvulsant	*Staphylococcus aureus*	Disc diffusion (0.78–6.25 mg/mL)	[70]
*Inga vera* Willd.	Phenols, flavonoids, tannins, saponins, anthraquinones, alkaloids, terpenes	Antimicrobial	*Escherichia coli*, *Klebsiella pneumoniae*, *Staphylococcus aureus*, *Pseudomona aeruginosa*, and *Candida albicans*	Disc diffusion (35 μg/mL)	[23]
*Leucaena leucocephala* (Lam.) de Wit	Essential oils	Central nervous system depressant, anthelmintic, and antidiabetic	*Staphylococcus aureus*, *Esherichia coli*, *Bacillus subtilis* and *Pseudomonas aeruginosa*, *Aspergillus niger*, *Rhizopus stolon*, *Penicillium notatum* and *Candida albicans*	Microdilution (100 μg/mL, 50 μg/mL, 25 μg/mL, 12.5 μg/mL)	[71]
*Lonchocarpus punctatus* Kunth	Alkaloids, camptothecins, epipodophyllotoxins, and taxanes	Anticancer	Cell model	Colorimetric assay	[32]
*Lysiloma acapulcense* (Kunth) Benth.	Tannins	Antimicrobial	*E. coli*, *P. aeruginosa*, *S. aureus* and *C. albicans*	Well diffusion (2.5 µg/mL to 5.0 µg/mL)	[72]
*Macroptilium lathyroides* (L.) Urb.	Flavonoids, polyphenols, terpenoids, saponins, and alkaloids	Antioxidant, antibacterial, cytotoxic, anticancer, and antifungal.	*Staphylococcus aureus* and *Escherichia coli*	Disc diffusion (1000 µg/mL, 750 µg/mL, and 500 µg/mL)	[73]
*Mimosa malacophylla* A.Gray	Phenols, tannins, flavonoids	Not reported	*Stenotrophomonas maltophilia*	Well diffusion (2.9 ± 0.5 mg/mL^−1^)	[74]
*Mucuna pruriens* (L.) DC.	Phenols, tannins	Astringent, laxative, anthelmintic, alexipharmic, and tonic	*Staphylococcus aureus*, *Escherichia coli*, *Bacillus subtilis*, *Pseudomonas aeruginosa*	Well diffusion (240 mg/mL)	[24]
*Neltuma glandulosa* (Torr.) Britton & Rose	Alkaloids	Antibacterial, antifungal, anti-infective, and antiparasitic activity	*Leishmania donovani*, *Plasmodium falciparum*, *Cryptococcus neoformans*, *Mycobacterium intracellulare*	Microdilution (0.66–20 μg/mL)	[33]
*Neltuma juliflora* (Sw.) Raf.	Alkaloids	Antibacterial	*Staphylococcus aureus*, *Staphylococcus epidermidis*, *Escherichia coli* and *Pseudomonas aeruginosa*	Broth dilution (2.5 mg/mL)	[25]
*Neltuma laevigata* (Humb. & Bonpl. ex Willd.) Britton & Rose	Phenols and alkaloids	Antimicrobial and antioxidant	*Staphylococcus aureus*, *Escherichia coli*, *Candida tropicalis* and *Fusarium moniliforme*	Broth dilution (0.08–4.62 mg/mL)	[60]
*Neptunia oleracea* Lour.	Alkaloids, glycosides, flavonoids, proteins, terpenoids, phytosterols, and tannins	Antioxidants and anti-inflammatory	*Staphylococcus aureus*, *Escherichia coli*, *Pseudomonas aeruginosa* and *Candida albicans*	Disc diffusion (10–100 mg/mL)	[35]
*Pachyrhizus erosus* (L.) Urb.	Isoflavones	Antifungal	*Colletotrichum gloeosporioides*, *Fusarium oxysporum*, and *Rhizopus stolonifer*	Disc diffusion (0.5–250 µg/mL)	[14]
*Parkinsonia aculeata* L.	Alkaloids, glycosides, flavonoids, terpenoids, and tannins	Antibacterial	*Staphylococcus aureus*, *Escherichia coli*, and *Pseudomonas aeruginosa*	Disc diffusion (12.5–50 mg/mL)	[75]
*Parkinsonia florida* (Benth. ex A.Gray) S.Watson	Alkaloids, carbohydrates, saponins, phenols, flavonoids, proteins, cardiac glycosides	Antibacterial	*Staphylococcus aureus* and *Escherichia coli.*	Disc diffusion (125–2000 µg/mL)	[18]
*Parkinsonia praecox* (Ruiz & Pav.) Hawkins	Triterpenes	Anticancer, antibacterial	*Listeria monocytogenes*	Microdilution (2000 µg/mL)	[19]
*Phaseolus coccineus* L.	Lectins	Antinoplastic and antifungal.	*Candida albicans*, *Penicillium italicum*, *Helminthosporium maydis*, *Sclerotinia sclerotiorum*, *Gibberalla sanbinetti* and *Rhizoctonia solani*	Disc diffusion (31.3–250 mg/mL)	[76]
*Phaseolus lunatus* L.	Isolated and hydrolyzed proteins	Antibacterial, antioxidant, anti-inflammatory	*Staphylococcus aureus*, *Escherichia coli*, *Bacillus cereus*, *Listeria monocytogenes* and *Pseudomonas aeruginosa*	Well diffusion (500, 375, 250, 200, and 150 mg/mL)	[77]
*Phaseolus vulgaris* L.	Lectins	Antibacterial and antifungal	*Staphylococcus aureus*, and *Streptococcus mutants*, *Pseudomonas aeruginosa* and *Klebsiella pneumonia*	Microdilution (0.24–1000 μg/mL)	[36]
*Pithecellobium dulce* (Roxb.) Benth.	Alkaloids, anthraquinones, flavonoids, cardiac glycosides, proteins, tannins, sugars, and terpenoids.	Anti-inflammatory, antivenom, protease inhibitor, spermicide, antimicrobial, and antituberculosis activity	*Bacillus subtilis*, *Enterococcus faecalis*, *Micrococcus luteus*, *Staphylococcus aureus* and *Staphylococcus epidermidis)*, *Aeromonas hydrophila*, *Alcaligenes faecalis*, *Enterobacter aerogenes*, *Escherichia coli*, *Klebsiella pneumoniae*, *Pseudomonas aeruginosa* and *Salmonella typhimurium*	Microdilution (200–1000 µg/mL)	[37]
*Rhynchosia minima* (L.) DC.	Essential oils	Antimicrobianas and antioxidantes	*Acenotobacter calcoacetilus*, *Bacillus subtilis*, *Citrobacter freundii*, *Escherichia coli*, *Proteus vulgaris*, *Pseudomonas aeruginosa*, *Salmonella typhii*, *Staphylococcus aureus* and *Yersinia enterocolitica.*	Well diffusion (100 µg/mL)	[78]
*Senegalia berlandieri* (Benth.) Britton & Rose	Phenols, tannins, diterpenes, sterols, triterpenes, and saponins	Antibacterial	Cell model	Disc diffusion (100 mg/mL)	[79]
*Senegalia greggii* (A.Gray) Britton & Rose	Phenols, tannins, diterpenes, sterols, triterpenes, and saponins	Antibacterial	Cell model	Disc diffusion (100 mg/mL)	[79]
*Senna crotalarioides* (Kunth) H.S.Irwin & Barneby	Triterpenes, alcohols, and phytosterols	Anti-inflammatory	Cell model	No data recorded	[80]
*Senna hirsuta* (L.) H.S.Irwin & Barneby	Essential oils	Antimicrobial	*Escherichia coli*, *Staphylococcus aureus*, *Bacillus subtilis* and *Aspergillus niger*	Microdilution (78–625 μg/mL)	[48]
*Senna obtusifolia* (L.) H.S.Irwin & Barneby	Saponins, tannins, alkaloids, and flavonoids.	Antimicrobial	*Neisseria gonorrheae*, *Salmonella* sp., *Pseudomonas aeruginosa*, *Proteus vulgari*, *Staphylococcus aureus* and *Streptococcus aerugenosa*	Disc diffusion (200–1000 μg/mL)	[47]
*Senna occidentalis* (L.) Link	Tannins, alkaloids, glycosides, flavonoids, steroids, saponins, anthraquinones, and flobanoids	Antimalarial, antitrypanosomal, immunosuppressive, anti-inflammatory, larvicidal, antidiabetic, anticancer, antiulcer, and hepatoprotective.	*Escherichia coli*, *Klebsiella pneumoniae*, *Candida albicans*, *Staphylococcus aureus*, *Pseudimonas aeruginosa* and *Salmonella typhi*	Well diffusion (80 and 120 mg/mL)	[40]
*Senna septemtrionalis* (Viv.) H.S.Irwin & Barneby	Raw extracts	Diuretic activity and neuropharmacological effects	Neuropharmacological effects	No data recorded	[38]
*Senna wislizeni* (A.Gray) H.S.Irwin & Barneby	Flavonols	Laxative, antimicrobial, antiviral, antifungal, anti-inflammatory, antitumor, antioxidant	*Escherichia coli* and *Salmonella thyphimurium*	Agar overlay bioautography	[15]
*Sophora tomentosa* L.	Hydrocarbons, sterols, terpenes	Antioxidants, antimicrobials, anti-inflammatories, and anticancer agents	*Bacillus subtilis*, *S. aureus* and *E. coli*	Well diffusion (50 mg/mL)	[27]
*Tephrosia cinerea* (L.) Pers.	Phenols	Antimicrobial	*Pseudomonas aeruginosa*, *E. coli*	Broth dilution (10–90 mg/mL)	[59]
*Vachellia farnesiana* (L.) Wight & Arn.	Phenols, tannins, diterpenes, sterols, triterpenes, and saponins	Antibacterial	*Providencia alcalifaciens*, *Micrococcus roseus*	Disc diffusion (100 mg/mL)	[79]
*Vachellia rigidula* (Benth.) Seigler & Ebinger	Phenols, tannins, diterpenes, sterols, triterpenes, and saponins	Antibacterial	*Providencia alcalifaciens*, *Micrococcus roseus*	Disc diffusion (100 mg/mL)	[79]
*Vigna luteola* (Jacq.) Benth.	Flavonoids and isoflavonoids	Antioxidant, antifungal, antitumor, antiparasitic, hypoglycemic, hepatoprotective, renal protection, antibacterial, hypotensive, and hypolipidemic	Cell model	No data recorded	[81]
*Vigna vexillata* (L.) A.Rich.	Sterols and isoflavones	Hypoglycemia, antihypertensive, cholesterol-lowering, antioxidant, antibacterial, anticancer	Cell model	No data recorded	[39]
*Zapoteca portoricensis* (Jacq.) H.M.Hern.	Alkaloids, saponins, tannins, terpenoids, flavonoids	Antimicrobial, antiviral, antioxidant	*S. aureus*, *Streptococcus pyogenes*, *E. coli*, *K. pneumoniae*, *P. aeruginosa*, *C. albicans*, *Microsporum audouinii*	Disc diffusion (5.0, 10.0, 20.0 mg/mL)	[28]
*Zornia diphylla* (L.) Pers.	Essential oils	Antifungal, antimicrobial	*Salmonella typh*	Microdilution (50 µg/mL)	[45]

## Data Availability

The data presented in this study are available within the article.
